# Locust Dynamics: Behavioral Phase Change and Swarming

**DOI:** 10.1371/journal.pcbi.1002642

**Published:** 2012-08-16

**Authors:** Chad M. Topaz, Maria R. D'Orsogna, Leah Edelstein-Keshet, Andrew J. Bernoff

**Affiliations:** 1 Department of Mathematics, Statistics, and Computer Science, Macalester College, Saint Paul, Minnesota, United States of America; 2 Department of Mathematics, California State University at Northridge, Los Angeles, California, United States of America; 3 Department of Mathematics, University of British Columbia, Vancouver, British Columbia, Canada; 4 Department of Mathematics, Harvey Mudd College, Claremont, California, United States of America; The University of Sydney, Australia

## Abstract

Locusts exhibit two interconvertible behavioral phases, solitarious and gregarious. While solitarious individuals are repelled from other locusts, gregarious insects are attracted to conspecifics and can form large aggregations such as marching hopper bands. Numerous biological experiments at the individual level have shown how crowding biases conversion towards the gregarious form. To understand the formation of marching locust hopper bands, we study phase change at the collective level, and in a quantitative framework. Specifically, we construct a partial integrodifferential equation model incorporating the interplay between phase change and spatial movement at the individual level in order to predict the dynamics of hopper band formation at the population level. Stability analysis of our model reveals conditions for an outbreak, characterized by a large scale transition to the gregarious phase. A model reduction enables quantification of the temporal dynamics of each phase, of the proportion of the population that will eventually gregarize, and of the time scale for this to occur. Numerical simulations provide descriptions of the aggregation's structure and reveal transiently traveling clumps of gregarious insects. Our predictions of aggregation and mass gregarization suggest several possible future biological experiments.

## Author Summary

Locusts such as *Schistocerca gregaria*, *Locusta migratoria*, and *Chortoceites terminifera* periodically form highly destructive plagues responsible for billions of dollars in crop losses in Africa, the Middle East, Asia, and Australia. These locusts usually exist in the so-called solitarious behavioral phase and seek isolation; gregarious individuals, however, are attracted to conspecifics. Previous experimental work has uncovered the causes of phase change in individual insects: principally, sustained exposure to sparse or crowded conditions. An open problem is to understand the intrinsic roles that phase change and social interaction play in the transition from an initially disperse, solitarious population to an aggregated, destructive marching hopper band of gregarious individuals. To this end, we construct a mathematical model that describes the interplay of phase change and spatial dynamics. Through analysis and numerical simulations, we determine a critical density threshold for gregarious band formation and quantify the collective phase change over time. We also discuss implications of our work for preventative management strategies and for possible future biological experiments.

## Introduction

Outbreaks of locusts such as *Schistocerca gregaria*, *Locusta migratoria*, and *Chortoceites terminifera* regularly afflict vast areas of Northern Africa, the Middle East, Asia, and Australia. Depending on climate and vegetation conditions, billions of voracious locusts aggregate into destructive swarms that span areas up to a thousand square kilometers. A flying locust swarm can travel a few hundred kilometers per day, stripping most of the vegetation in its path [1]–[4]. A recent locust plague in West Africa (2003–2005) severely disrupted agriculture, destroying $2.5 billion in crops destined for both subsistence and export. Despite control efforts totalling $400 million, loss rates exceeded 50% in certain regions [5], [6]. These numbers alone attest to the urgency of finding better ways to predict, manage, and control locust outbreaks.

Between outbreaks, locusts are mainly antisocial creatures who live in arid regions, laying eggs in breeding grounds lush with vegetation. Resource abundance may, on occasion, support numerous hatchings, leading to a high population density. Overcrowding at resource sites promotes transition to a social state in a self-reinforcing process. The social locust nymphs may display mass migration behavior. Within the newly formed group, individuals cohere via sensory communication, whether visual, chemical, and/or mechanical [3]. Outbreaks may be exacerbated in periods of drought, when large numbers of locusts congregate on the same breeding or feeding grounds [7]–[9].

Locusts are *phase polyphenic:* while sharing the same genotype, individuals may display different phenotypes [10], [11] that incorporate variations in morphology [12], coloration [13], reproductive features [14] and, significantly, behavior [15], [16]. An individual can change from a *solitarious* state (preferring isolation) to a *gregarious* one (seeking conspecifics). Behavioral state is plastic [3], [11], [15] and strongly dependent on local population density: in sparse surroundings, a gregarious locust transitions to the solitarious state [15] and vice versa in crowded environments. These phase transitions are called solitarization and gregarization. Gregarization dominates when large numbers of locusts gather at the same site, potentially leading to a destructive outbreak [8], [9].

Locust gregarization may be induced by visual, olfactory, or tactile cues. For the desert locust *Schistocerca gregaria*, the most potent stimulus is tactile: repetitive stroking of the femora of hind legs [15]–[17] functions as a crowding indicator. Mechanosensory stimulation of leg nerves leads to serotonin cascades in the metathoracic ganglion, and initiates gregarious behavior [16]–[18]. Gregarization can be induced by rubbing a locust's hind leg for 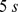 per minute during a period of 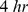 [17]. Cessation of physical contact leads to solitarization after 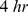, though the degree of solitarization achieved during that time depends on the individual's ancestry.

Experiments and models have shed much light on how group alignment [19]–[22] and group motion [23], [24] depend on group size or density and treatments such as diet and denervation. For instance, a low-protein diet (which motivates cannibalism in locusts) leads to stronger interactions between individuals and lowers the threshold density beyond which mean speed and group coherence increase [24]. Other data-driven studies include models based on a well-known physics paradigm for self-propelled particles [25] and explore the transition between a disordered and a coherent marching group. Both [26] and [27] study the dynamics of rolling patterns formed by flying, gregarious swarms. A logistic map was introduced in [28] to describe phase change via a birth rate and a carrying capacity dependent on population density modulated by stochastic effects.

Our current work complements previous locust modeling studies in several ways. First, many of the previous models are individual-based (Lagrangian) simulations, where the position, velocity, and interactions of individual locusts are tracked [19], [20], [22]–[24]. Ours is density-based (Eulerian), allowing techniques of partial differential equations (PDEs) and their extensions (integro-PDEs) to be utilized. Second, we concentrate on gregarious-solitarious transitions not yet explicitly considered in [20], [24]. We address intrinsic attractive-repulsive social interactions, whereas many current models consider interactions with clumped resources and environmental heterogeneity as their focal points [8], [9]. Finally, some models [24] include anisotropic interactions such as different responses to anterior and posterior neighbors, or consider Newtonian dynamics. To explore minimal mechanisms sufficient for band formation, our work instead uses isotropic interactions and a kinematic approach. The open problem we address via mathematical modeling is to quantify and describe collective gregarization, a key, early process that necessarily occurs before the emergence of a destructive locust outbreak. We do this by linking the physiology of individual-level phase change and interphase interactions to predictions at the level of the gregarious hopper band as a whole.

We investigate the onset of an outbreak by constructing a continuum mathematical model of behavioral phase for interacting gregarious and solitarious locusts. We classify and quantify group dynamics in wide swaths of parameter space, a task which is challenging by numerical techniques alone. We find that in the limit of low densities, both phases are uniformly spread and the solitarious phase dominates. For sufficiently large populations, a dense, traveling patch of gregarious locusts suddenly emerges, while solitarious locusts become more and more scarce. We identify locust clustering at high densities with the onset of a hopper band. Through analysis of our model, we calculate the critical density beyond which the gregarious group forms, and for the final ratio of gregarious to solitarious locusts. We determine these quantities in terms of behavioral parameters at the level of individual locusts, hence connecting individual and group properties. Our model also displays population-level hysteresis, which has implications for locust management.

## Model

### Model construction

Locusts in a group are subject to attractive and/or repulsive forces based on combined sensory, chemical, and mechanical cues that affect their motion. We assume that sensing is directionally isotropic, a reasonable approximation [29] for organisms receiving sensory inputs of a variety of types, although directional models are possible as well [30]. Rather than tracking individual locusts, we consider a population density field 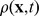 moving at velocity 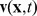. Continuum population modeling [31], [32] allows us to apply analytical tools in order to characterize swarm formation and structure. Our work draws from classic swarm modeling in which a conserved population density field 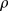 moves at a velocity 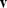 that arises from social interactions:
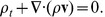
(1)This is the well-known mass balance equation that tracks individuals moving collectively at velocity 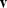. It is typically assumed that individuals can sense the population density nearby, and that this sensing gives rise to attractive-repulsive social forces 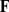, or alternatively, social potentials 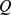 (the negative gradients of which are forces). Within this context, the contribution 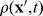 of a small clump of individuals at location 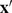 to the force on the individual at position 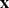 is given by 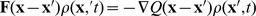. The corresponding velocity is proportional to the forces exerted by neighbors at all spatial locations, so that 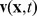 is given by integration over all 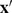 as
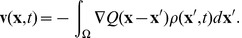
(2)The expression for the velocity 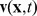 in Eqn. (2) is a convolution of the density 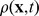 and the social interaction force 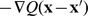, which describes the influence of the locust population at location 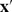 on that at location 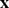. This is a common formulation of so-called *nonlocal* interaction models [33]–[36], which capture interactions that are spatially distributed, in contrast to pure partial differential equations, which include only local terms such as derivatives and gradients, and which describe interactions only over infinitesimal ranges. Nonlocal aggregation models have been studied for various social interactions 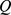; known solutions include steady swarms, spreading populations, and contracting groups. We use the notation 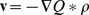 to denote the convolution in Eqn. (2). We assume that 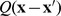 is radially symmetric and depends only on the distance between 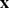 and 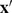. The detailed forms of 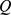 in the case of solitarious and gregarious locusts will be described later.

To adapt Eqs. (1) and (2) to biphasic insects, we introduce separate density fields for solitarious and gregarious locusts, 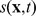 and 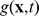, respectively, and the total local density 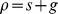. With marching locusts in mind, we consider a two-dimensional geometry, with 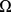 representing the spatial domain and 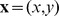 as spatial coordinates. We now include the phase transitions between solitarious and gregarious locusts. To do so, we define two density-dependent functions, 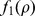 for the the rate of gregarious-to-solitarious transition, and 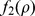 for the rate of solitarious-to-gregarious transition. Our model thus reads
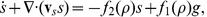
(3a)

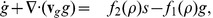
(3b)where the velocities are given by
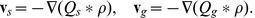
(4)These equations are complete once we specify the solitarious and gregarious social interactions 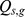 and the density-dependent conversion rates 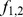. Since solitarious locusts are crowd-avoiding, we take 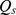 to be purely repulsive. Gregarious locusts, on the other hand, are attracted to others, except for short-distance repulsion due to excluded volume effects. Hence, we model 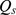 and 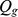 as
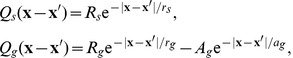
(5)where 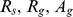 are interaction amplitudes that determine the strengths of attraction and repulsion, and 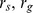 and 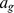 are interaction length scales that represent typical distances over which one locust can sense and respond to another.

The above forms of 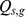 describe social interactions that decay exponentially away with distance from the sensing individual and are chosen to be isotropic for simplicity. As evident from Eqn. (5), 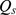 is purely repulsive for all choices of 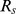 and 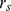. On the other hand, 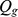 is the difference of two exponentials, implying that there may be a distance at which repulsion and attraction balance, resulting in no net contribution to the velocity. The location of this balance point can be obtained by imposing 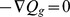 to obtain the critical distance
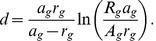
(6)Depending on the choice of social interaction parameters, the expression for 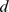 may yield unphysical results such as negative distances. The distance 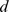 also pertains only to two isolated locations 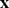 and 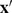 and does not capture population-level features. Even for meaningful values of 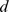, a collection of individuals interacting under 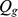 may disperse, aggregate, or clump. It is thus important to choose the appropriate parameter ranges for 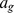, 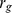, 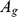 and 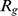 so that the tendency of gregarious locusts to aggregate is modeled properly. Mathematical studies have shown that in order for cohesiveness to occur, the parameters in 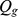 must lie in a particular regime that leads to clumping [37]. Thus, we require 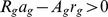 so that repulsion dominates at short length scales, and 
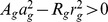
 so that attraction dominates at longer ones. Taken together, these conditions guarantee a meaningful critical distance 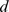 and macroscopic clumping behavior. We assume these conditions to hold for the remainder of this paper.

It remains to specify how density affects transitions from one phase to another. We call upon the biological observation that at higher densities, gregarization proceeds more quickly and solitarization more slowly. We model the phase conversion rates with the rational functions
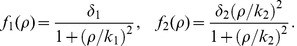
(7)The parameters 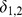 are maximal phase transition rates and 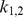 are characteristic locust densities at which 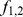 take on half of their maximal values. Note that 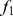 decreases with 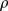, capturing the inverse relationship between solitarization rate and density, while 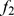 increases with 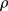 and saturates at 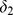, describing speedier gregarization at higher densities.

Our complete model consists of Eqs. (3)–(7) together with initial conditions specifying 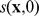 and 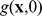. We consider a spatially periodic domain, which simplifies both numerical simulation and mathematical analysis. In certain laboratory studies using ring-shaped arenas, such boundaries are natural (while being less ideal for comparison with field studies) [20]. We do not include locust reproduction or death as these occur on much longer time scales than phase change.

The model presented here is a general one containing some fundamental elements of locust dynamics. This work can be readily modified and extended to include details pertaining to different locust species, interactions with the surrounding environment, locust reproduction, and more. For instance, in our model, we have not explicitly accounted for the differing activity levels of solitarious and gregarious individuals [11]. Additionally, while gregarization is relatively fast for *Schistocerca gregaria*, full solitarization may occur only after several generations of locusts. The phase conversions of *Chortoicetes terminifera*, on the other hand, are characterized by similar timescales for the two phase conversions, so that both gregarization and solitarization occur rapidly within the lifetime of a single locust individual [38]. On another note, vegetation or waterway patterns may impose spatial inhomogeneities such as non-uniform initial distributions of solitarious locusts, or attraction to preferred sites. Preexisting models in the literature have pointed out the important link between the spatial distribution of vegetation, as well as nutritional quality, on locust clustering, gregarization, and swarming [8], [9], [39], [40]. All of these elements could be used to refine our model for predictive purposes. However, as the first work in the continuum modeling of locust population phase change, ours begins with the fundamental model contained in Eqs. (3)–(7). Our model is complementary to the preexisting ones in that we focus on how inherent inter-individual interactions can lead to gregarization and swarming, even in a spatially homogeneous environment. Multi-generational dynamics, differential activity levels, resource distribution, and related factors could be considered as possible extensions of our work.

### Parameter selection

Some of our results are analytical formulas, which may be evaluated for any desired parameter values. Other results depend on numerical computations, and these require specific choices of parameters. For these results, we consider two different sets of phase transition parameters. (1) Most of our numerical results have been obtained using our *default set* of parameters, based on estimates from the biological literature. Specifically we take 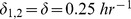, corresponding to a gregarization time scale of approximately 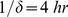 for desert locusts (for whom some – but not total – solitarization occurs on the same time scale) [11], [17]. We also take 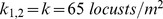, since for desert locusts, the critical density for the onset of collective motion is 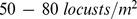 [24]. We will allow for some deviation from 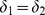 and 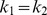 via a parameter sensitivity analysis. (2) To examine situations with large differences in the rates of gregarization and solitarization, we consider an *alternative set* of parameters with 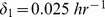 and 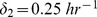, so that gregarization is an order of magnitude faster that solitarization. We take 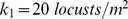 and 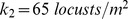 to model a gregarious-to-solitarious transition that occurs at a higher density threshold than the solitarious-to-gregarious transition.

We use the same social interaction parameters for all results (variations from this set are accounted for by a sensitivity analysis). To estimate the social interaction length scale parameters in Eqs. (5), we apply the results of [20], [24], which identify the “sensing range” of a desert locust as 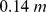, and the “repulsion range” as 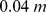, close to the approximately 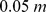 body length of a desert locust at the fifth instar of its development. For the gregarious phase we thus set the repulsion length scale at 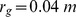 and the attractive one at 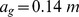, corresponding to the experimental sensing range. These choices agree with theoretical studies showing that for cohesive swarms, attraction occurs over longer length scales than repulsion [33], [41]. We also assume that solitarious locusts are repelled from others at their sensing range, so that 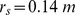. These choices satisfy 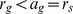 which is assumed for the remainder of this paper.

Finally, we estimate 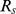, 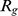, and 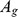 via explicit velocity computations. The speed of a locust when it is alone varies between 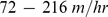, depending on diet [24]. At the upper end, this is roughly one body length per second. When it is moving in a group, the individual's speed varies in a tighter range of 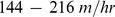 [24]. In making our phase-dependent velocity estimates, we interpreted the “moving alone” and “moving in a group” data as typical to solitarious and gregarious locusts, respectively. Using these biological measurements and Eqn. (4), we find 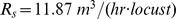, 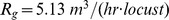, and 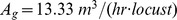. Details are given in Text S1. Our choices of social interaction parameters satisfy conditions mentioned in the previous section, namely 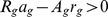, and 
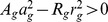
 so that gregarious insects will clump.

Most of our parameter choices have been inferred or estimated from published laboratory experiments. It is possible however, that in the field, some parameter values may be quite different from the ones we have used. For instance, locusts in the field may pause while marching to perch on the vegetation, giving rise to an effective speed that is lower than what measured in lab experiments, where perching does not occur. It is also noteworthy that gregarious locusts are more active than solitarious locusts, a fact that is reflected by our method of choosing 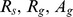 from estimates of the velocities of individuals when moving alone and in a group. As we describe below, we analyze our model varying all parameters within reasonable bounds: our results are qualitatively the same.

## Results

We first determine the simplest solutions to the model, namely those for which the densities of gregarious and solitarious locusts are in a spatially uniform steady state. We probe the stability of that uniform state using linear stability analysis (LSA), a calculation that addresses whether small, spatially nonuniform perturbations grow or decay. This is equivalent to determining the signs of eigenvalues of the linearized system, where positive (negative) eigenvalues imply growing (decaying) perturbations. The rate of initial growth/decay depends on the wavenumber of the perturbation. The growing perturbations can be interpreted in terms of nascent aggregates of locusts, and the wave numbers as the number of aggregates per unit area. The analysis provides a condition for the onset of aggregation, namely the emergence of positive eigenvalues of the linearized model. In our case, this aggregation condition is shown below in Eqn. (14). LSA cannot, in general, predict the ensuing dynamics once perturbations have grown to a large size. Further analysis uses an approximation to eliminate the spatial dependence of the model, which enables an analytical prediction of the proportion of solitarious and gregarious locusts on a longer time scale. To visualize the dynamics of aggregation, we perform numerical simulations in one spatial dimension using the linear stability analysis to identify regimes of interesting behavior. The model displays population-level hysteresis.

### Homogeneous steady states

**Figure 1:**
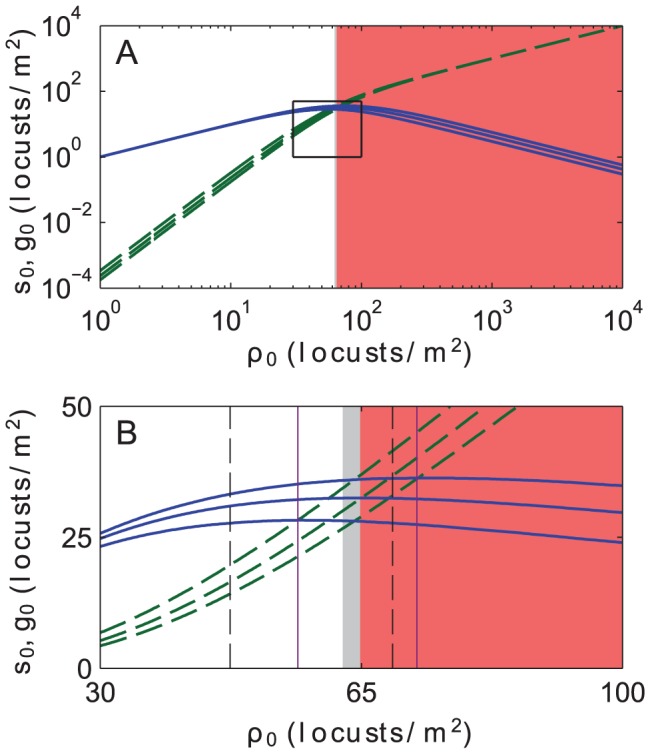
Spatially homogeneous steady states (HSS). (A) Spatially homogeneous solitarious (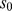, solid blue) and gregarious (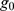, broken green) steady state locust density as the total locust density (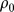) is varied. For each set of curves, the middle curve represents the solution for our default phase change parameters, 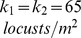 and 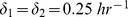. The bottom and top curve in each set show parameter sensitivity; they are the 25th and 75th percentile values for 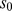 and 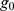 over 10,000 parameter sets of 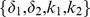 sampled from uniform distributions centered at the default values and varying by 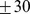
%. In both the thin grey and red regions, the HSS is linearly unstable to small perturbations. Additionally, in the red region, 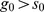, while in the grey and white regions, the opposite holds. (B) A blow-up of the boxed transition region in (A) around which the value of 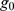 overtakes 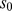. The dashed black vertical lines indicate the 25th and 75th percentile for this transition. The solid purple vertical lines indicate the 25th and 75th percentile values for the onset of linear instability.

The solitarious 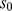 and gregarious 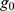 homogeneous steady-state (HSS) solutions of Eqn. (3) can be written in terms of the total uniform density 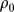, which is simply the mean value of 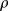 for a specified initial condition. The full expressions for 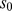 and 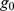 in terms of 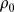 appear in Text S1; in the small 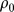 limit these are approximately
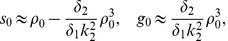
(8)while in the limit of large 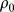 we find
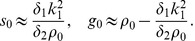
(9)The low density HSS is thus composed mostly of solitarious locusts and vice versa for the high-density case, showing the non-monotonicity of 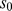 with respect to total density 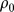. In Fig. 1(A) we plot the HSS 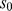 (middle solid blue curve) and 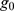 (middle broken green curve) for our default set of phase change parameters, 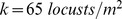 and 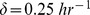.

As shown, 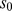 initially increases with 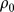. At a critical density 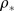, 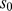 reaches a maximum, whereas 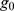 keeps increasing monotonically. Fig. 1(B) shows a blow-up of the region near 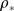. For our default parameters, the maximum value 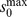 is attained at 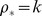, the same density value for which solitarious and gregarious densities coincide so that 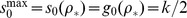. However, this feature is a result of our choice 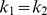 and 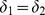. In general, the point of maximum solitarious density and the point of equal solitarious and gregarious density do not coincide, as is directly deducible from the full expressions for 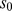 and 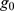 in Text S1. To give a sense of detuning from our parameter estimates, we also calculate and plot 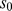 and 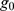 for parameter sets chosen randomly from uniform distributions centered at our estimated default set of values for 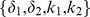. The bottom and top curve in each set show the 25th and 75th percentile values.

We also study a much more general case where 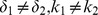, in keeping with the distinct rates of transition and critical transition densities seen biologically. As an alternative way to understand the HSS solutions, we consider the fractions 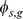 of solitarious and gregarious locusts, where 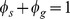. As shown in Text S1, for the HSS,
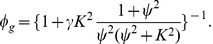
(10)Here, 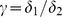 is the ratio of maximal solitarization rate to maximal gregarization rate, 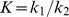 is the ratio of the characteristic solitarization and gregarization densities for individuals, and 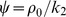 is a rescaled spatially homogeneous density. The gregarious fraction 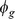 is monotonically increasing in 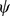, and hence in 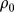; that is to say, as total density increases, the gregarious fraction increases. For small 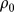, 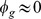, but as 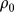 increases, there is a crossover between solitarious and gregarious populations. Uniformly spread solitarious populations cannot be sustained when the density is too high: the gregarious state will necessarily become the dominant one.

### Linear stability analysis

To determine conditions under which a nearly uniformly spread locust population aggregates or disperses, we study the linear stability of the HSS (details appear in Text S1). The calculation is a standard but somewhat tedious exercise. In nonlocal systems such as ours, linear stability results depend on the Fourier transforms 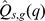 of the interaction potentials 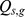. For our locust model, the stability of the HSS depends on the eigenvalue
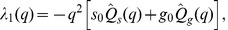
(11)where 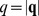 is the perturbation wave number and the Fourier transforms 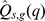 in two dimensions are
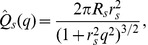
(12)

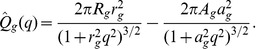
(13)Observe that the eigenvalue 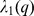 depends on all of the individual-based parameters governing rates of phase change (via 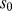 and 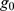) and all of the social interaction amplitudes and length sensing length scales. The HSS derived in the previous section is stable to small perturbations if 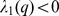 for all 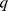. If 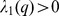 for some 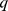, then the HSS is unstable to perturbations of those wave numbers.

**Figure 2:**
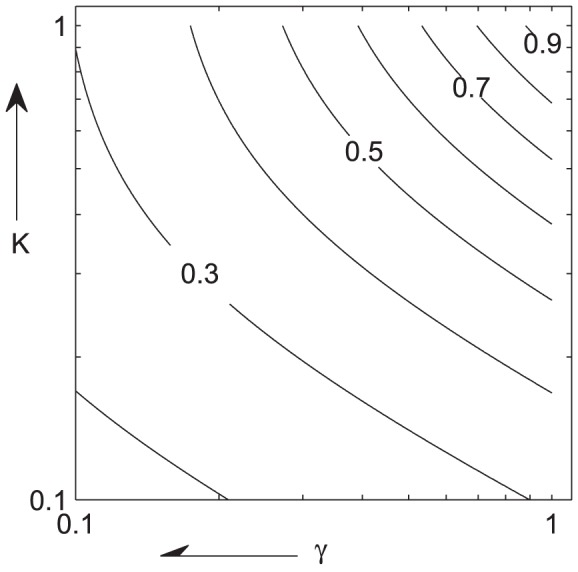
Linear stability of spatially homogeneous steady state (HSS) solutions. The dimensionless phase change parameter ratios 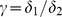 and 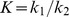 vary along the horizontal and vertical (we have used log axes). The contours indicate the critical value of rescaled density 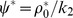. For rescaled densities greater than that value, the HSS solution is unstable. The critical rescaled density is monotonically increasing in both 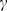 and 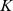. The arrow along the horizontal axis indicates the direction 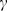 moves if the relative rate of gregarization is increased (faster gregarization). The arrow along the vertical axis indicates the direction 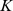 moves if the relative density threshold for gregarization is decreased (easier gregarization). For an accurate biological interpretation, one must multiply 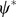 by 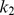 in order to obtain the unscaled critical density 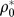.

Our full analysis of this eigenvalue appears in Text S1. We formulate the instability condition in terms of 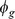,
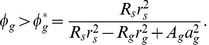
(14)If this condition is satisfied, initially small perturbations from the uniform steady state will grow. This inequality is a key result, and implies that if a sufficiently large fraction of the population is gregarious, the HSS solution is unstable. To obtain a more explicit condition in terms of the density 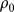, one must substitute 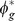 into Eqn. (10), which relates gregarious fraction to total (scaled) density. One may then calculate the critical density 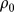 above which the HSS is unstable. Since 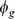 and 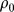 are monotonically related, we conclude that the HSS solution is unstable for sufficiently dense populations. The algebra is tedious, and relegated to Text S1. Instead, we present a contour plot in Fig. 2 which succinctly illustrates the stability features of the HSS. The phase change parameter ratios 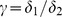 and 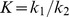 vary along the horizontal and vertical axes and the contours indicate the critical value of rescaled density 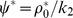. For scaled densities greater than 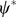, the HSS solution is unstable. The critical scaled density is monotonically increasing in both 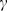 and 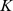. (Note that for an accurate biological interpretation, one must multiply 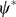 by 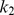 in order to obtain the unscaled critical density 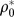.)

Upon inserting our default parameters in Eqn. (14) we find that the homogeneous solution is unstable for 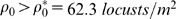. This value corresponds to the left border of the grey region in Fig. 1. For 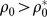, to the right of the border, we expect the onset of a locust hopper band, *i.e.*, formation of patches of high locust density that can seed the clustering and gregarization of other locusts. In Fig. 1, linear instability can occur even at densities 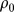 for which 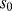 exceeds 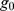 for our chosen parameters (represented by the center solid blue and center broken green curves). This result implies that the onset of instability leading to mass gregarization can take place even if solitarious locusts initially outnumber gregarious ones. We will later discuss mass gregarization in more detail. To visualize detuning from this set of parameters, we include the 25th and 75th percentile values of 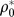 for onset of instability as vertical purple lines; these are again calculated by drawing 10,000 random samples of the parameters 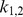, 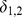, 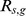, 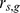, 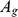, and 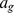. As seen from Fig. 1(b) our conclusions are robust across the randomly chosen parameter sets.

For our default set of biological parameters, 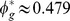 via Eqn. (14) and 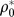 turns out to be near 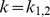. We stress that generically, it is not the case that 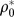 needs to be near 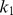 and/or 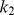. For our default parameter set, 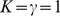, in which case 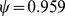, so that the critical value 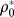 is 95.9% of 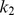, namely 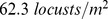. However, for different choices of 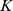 and 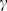, drastically different outcomes are possible. For instance, for our alternative parameter set where 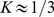 and 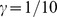, the critical density is 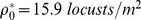, which is quite disparate from the individual gregarization density of 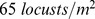, and is also less than the solitarization density of 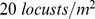. Furthermore, for different choices of the social interaction parameters entering into Eqn. (14), it is possible to obtain a critical gregarious fraction 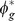 that is much less than 1/2, meaning that instability and clumping can occur even with just a few gregarious insects.

**Figure 3:**
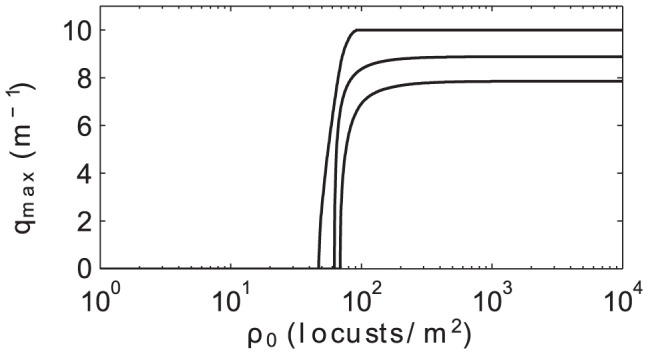
Maximally unstable perturbation wave number 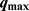 for homogeneous steady states with total density 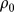. Similar to Fig. 1, the middle, bottom, and top curves show results for the 25th and 75th percentile as computed from 10,000 random parameter draws centered around our default parameter set. At low densities, there are no unstable perturbation wave numbers. Just past the critical density 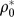, 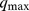 increases rapidly and then plateaus. For our default parameters, 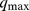 asymptotes to 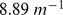 corresponding to a length scale of 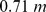.

For 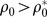, we can also find the wave number 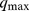 corresponding to the most rapidly growing perturbation. Fig. 3 shows 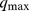 for our chosen parameters (center curve) as well as the 25th and 75th percentile values over the 10,000 random parameter draws. The most unstable wave number 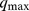 grows rapidly as a function of 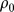 and then saturates at 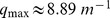, corresponding to a length scale 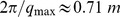 and indicating that the most quickly growing perturbations occur on the length scale of a few locust bodies.

Our linear stability analysis describes the behavior of small perturbations of uniform steady states, and is not expected to predict long-term or large-amplitude dynamics. For large perturbations, linear analysis is void. Additionally, even to analyze small perturbations of states other than uniform steady states, a different analysis would be needed.

### Numerical simulation

To illustrate the swarm dynamics described by Eqn. (3), we simulate the model on a one-dimensional periodic domain of length 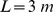 for a total population of 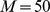 locusts. Periodicity of the domain is an important aspect of a robust numerical platform devised for these simulations: we exploit the fact that convolutions 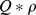 are easy to compute in Fourier space (where they are simply products, *i.e.*, 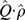), which significantly reduces the computational overhead. Computational issues associated with such convolutions also restrict us to one-dimensional simulations at present. At 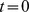 all locusts are solitarious and are randomly perturbed from the uniform density 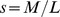, where 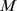 is the total population mass
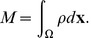
(15)We adjust some parameters so as to adapt our model to the one-dimensional case. Specifically, one must take square roots of 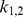 in order to collapse densities in a square to densities along a line segment. Consequently, for our default parameter set we choose 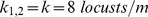 and 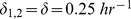, whereas for the alternative set we use 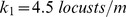, 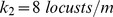, 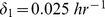 and 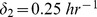. In both cases we take the interaction amplitudes 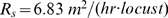, 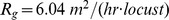, and 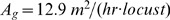, which have also been adapted from their original values to the one-dimensional case. The interaction length scales 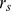, 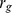, and 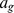 are the same as for the two-dimensional case. Details of the numerical method and the parameter choices appear in Text S1.

**Figure 4:**
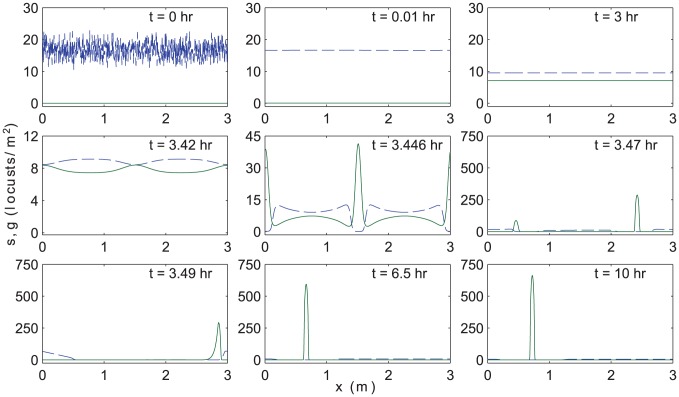
Numerical simulations of Eqs. (3). Snapshots depict the numerical solution of Eqs. (3)–(7) at different times 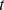 (in 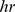) on a periodic domain of length 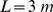 with the default set of phase change parameters. See also Fig. 5 for a comparison with the alternative parameter set. The solitarious (gregarious) density (in 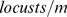) as a function of spatial position (in 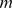) is shown in blue (green). The total population mass is 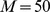 locusts and the initial condition is set at 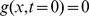 and 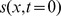 given by a random perturbation centered around 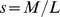. The top row of panels shows the fast smoothing of the initial state, and the subsequent evolution. Gregarization (approximately) occurs according to the spatially homogeneous version of Eqn. (3), as can be seen up until the second row of panels, where the small instability becomes significant. Two compactly supported clumps of gregarious locusts form, superposed on a very sparse population of solitarious insects. In the third row, the gregarious group travels as a propagating pulse, and eventually stops. During this stage, the gregarious and solitarious populations are essentially non-overlapping in space. As shown in Fig. 6, the group continues to slowly gregarize after it becomes stationary.

**Figure 5:**
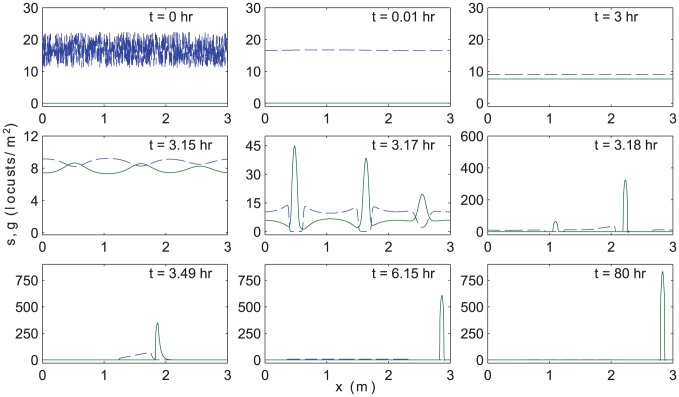
Numerical simulations of Eqs. (3). Similar to Fig. 4, snapshots at different times 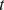 (in hours), but for the alternative set of phase change parameters. Note that three, rather than two clumps of gregarious locusts form at intermediate times. This simulation is continued until 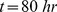 (last frame) to show the stability of the final cluster of gregarious locusts.

Results are shown in Fig. 4 for the default parameter set and in Fig. 5 for the alternative set. In each case, the snapshots show 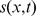 (dashed blue curve) and 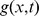 (solid green curve) at selected times. Starting from the randomized solitarious state at 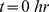, locusts rapidly redistribute to a roughly spatially uniform density until 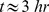. Tiny variations are present but not visible on the scales of these figures. Gregarization and subsequent rapid spatial segregation follow. In Fig. 4, between 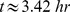 and 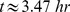, two compactly supported clumps of gregarious locusts emerge, superposed on a background of sparse, solitarious individuals. A similar transition occurs between 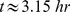 and 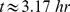 in Fig. 5, but for these parameter values, we find initial clustering with three, rather than two density peaks. The number (or alternatively, length scale) of transient clumps that form appears to be selected dynamically. This intermediate dynamical selection process and the coarsening that ensues are avenues for future numerical and analytical investigation. In each example, the disjoint clusters quickly merge due to the long-range attraction of gregarious individuals. A single remaining pulse is formed by 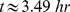 in both cases and travels until 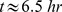, at which time the majority, but not all, of the solitarious locusts have transitioned to the gregarious form. Gregarization continues during the subsequent hours, albeit at a slower rate. For both figures, the gregarization of the final clump continues slowly, approaching an equilibrium at exponentially long times.

**Figure 6:**
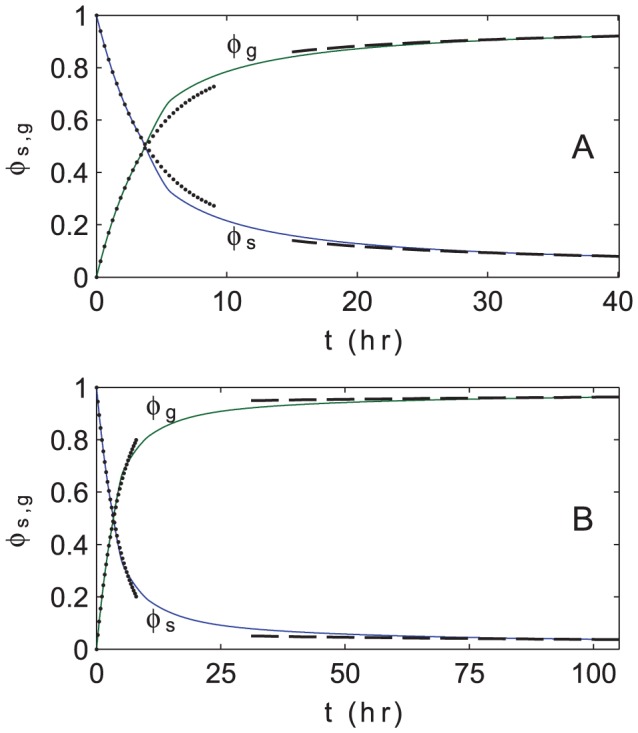
Population-level phase change over time. Mass fractions 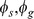 of solitarious (blue) and gregarious (green) locusts as a function of time (in hours) for the numerical simulations of Fig. 4 and Fig. 5. (A) Default set of phase change parameters, corresponding to the simulation in Fig. 4. (B) Alternative set of phase change parameters, corresponding to the simulation in Fig. 5. For both cases, at early times, these mass dynamics are well-approximated by the spatially homogeneous version of the governing equations Eqs. (3), whose solution, Eqn. (18), is shown as dotted curves. At late times, the mass dynamics are approximately described by the spatially segregated bulk theory of Eqn. (19), whose solution is shown as dashed curves.

To study the locust gregarization process further, we define the total mass of solitarious and gregarious locusts, 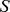 and 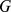, as
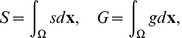
(16)so that the total population mass is 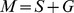. We also define the mass fractions
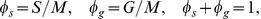
(17)which we before calculated for HSS solutions, but we now generalize for spatially varying states. These quantities will be useful to further our mathematical analysis. Fig. 6 shows 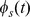 (blue curve) and 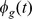 (green curve) as arising from the numerical simulations depicted in Fig. 4 and Fig. 5. Several distinct regimes are visible, and we discuss these below.

### Spatially-homogeneous and spatially-segregated bulk theories

As visible in the second and third panels of Fig. 4 and Fig. 5, the early-time dynamics of Eqs. (3) are approximately spatially homogeneous. As a result, spatially-dependent terms in Eqs. (3) are negligible, 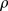 is approximately constant, and hence the governing equations are linear ordinary differential equations (ODEs) that are easily solved. We write the solution of these ODEs in terms of the mass fractions 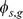,
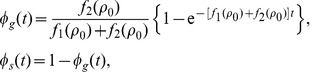
(18)where we have used the initial condition 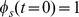. This analytical solution is plotted in Fig. 6 as a dotted line, and agrees closely with the numerical results for the first few hours.

In the later panels of Fig. 4 and Fig. 5, gregarious and solitarious locusts spatially segregate into areas with disjoint support. This means that in each distinct region, 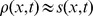 or 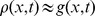. We thus consider a *bulk* model reduction to study the dynamics of the two non-overlapping solitarious and gregarious populations. In particular, we assume that solitarious locusts are spread throughout most of the domain 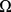, covering an area denoted 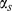, whereas gregarious locusts are confined to a region with area 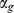. Within these areas, local densities are approximately 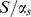 and 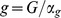. By integrating Eqs. (3) over the domain and assuming that 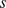 and 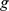 are approximately constant in their support, we obtain
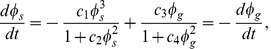
(19)where
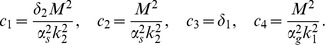
(20)The numerical solution of these ODEs (dashed lines in Fig. 6) agrees closely with the late time full-scale numerical simulation results, where we use values of 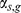 measured empirically from the terminal equilibrium. One can reduce Eqn. (19) to a single nonlinear ODE using 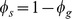, though this equation is not amenable to analytical solution. Since we are interested in the large population limit for which we expect potential large scale gregarization, we instead study Eqs. (19) for large 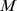. In this case, to leading order in 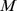, the bulk model reduces to
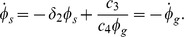
(21)Given the expressions for 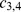 and the fact that 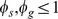, the first term is 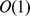 whereas the second one is much smaller, 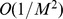. For large 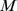 then, and to leading order, 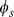 decays exponentially in time with rate 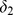. This result is based on the assumption of a segregated state, and thus would be expected to occur only once segregation is nearly complete.

Since for large 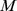 (nearly) the entire population will eventually become gregarious, the critical density 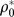 is a crucial result. If the population is in the stable regime (where 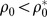) then mass gregarization can be avoided and solitarious and gregarious locusts can coexist as uniformly spread populations. However, as soon as the population shifts beyond the border of stability (where 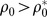) the group gregarizes and the onset of a locust hopper band is inevitable.

### Phase change and hysteresis

The biological literature discusses the importance of *hysteresis* in locust phase change, as reviewed, for instance, in [11]. It is important to disambiguate the possible meanings and interpretations of phase change hysteresis, to place this phenomenon within the context of our model, and most especially, to distinguish between hysteretic features at the individual and population levels.

One type of hysteresis is simply defined as “rates of gregarization [that] differ from rates of solitarization” [11]. Within our model, this type of hysteresis may be interpreted as cases where 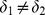 or 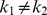. Our results thus far have accounted for this type of hysteresis in three ways. First, for our primary parameter set in which 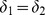 and 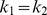, we have allowed deviations from equality by performing a sensitivity analysis incorporating variations of up to 30% from the base parameter values, as represented in the results of Fig. 1 and Fig. 3. Second, for our alternative parameter set, we have chosen 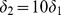 and 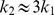. And finally, for analytical results such as the homogeneous steady states and their stability, we have obtained analytical formulas into which any values of 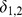 and 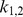 can be substituted.

Another interpretation of hysteresis relates to “solitarization [having] two phases: an initial rapid phase and a second, slower phase that requires insects to be maintained in isolation across successive moults – or generations” [11]. Our model is constructed on the time-scale of a single generation, and thus we cannot account for this type of hysteresis, which would require a multi-generational model.

**Figure 7:**
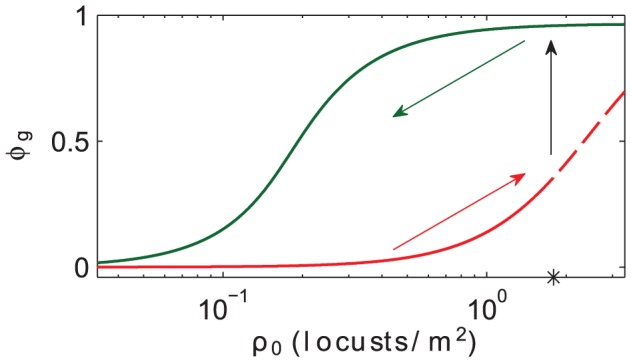
Population-level hysteresis as a function of average density 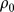. Gregarious mass fraction 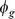 as the average density 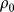 (total mass 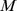 divided by domain length 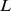) is varied as a control parameter. We use our alternative set of phase change and social interaction parameters, as in Fig. 5. The solid (dotted) red curve represents the stable (unstable) homogeneous steady state solution, as calculated via linear stability analysis. As 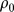 passes through the point of linear instability (marked with an asterisk) the solution jumps up to the green curve, which represents compactly supported gregarious aggregations obtained via numerical simulation, similar to the final states of Fig. 4 and Fig. 5. As 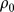 is decreased by slowly subtracting mass from aggregations on the green curve, the system remains on the upper branch even for values of 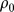 sufficiently small as to be in the regime where the uniform state is stable, thus demonstrating dynamical population-level hysteresis.

Finally, we can consider population-level hysteresis. In the context of our model, this type of hysteresis refers to macroscopic properties of solutions of Eqn. (3) (which are outputs of the model) as opposed to differences in individual-level parameters (which are inputs to the model) as in the first type of hysteresis described above. Numerical results suggest that our model has population-level hysteresis; see Fig. 7. This figure shows the gregarious mass fraction 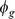 as the average density 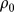 (total mass 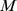 divided by domain length 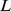) is varied as a control parameter. All phase change, social interaction, and physical domain parameters are the same as in Fig. 5.

The solid (dashed) red curve is an analytical result, representing the stable (unstable) HSS solution, as calculated previously via linear stability analysis. For small values of 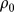, the HSS is stable to small perturbations. If locusts join the initially stable population the average density 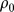 will increase (assuming a fixed spatial domain), shifting the uniform state to the right along the red curve; as yet no clustering will be evident. Beyond the point labeled with an asterisk, the uniform HSS loses stability and clustering occurs, as previously described. This corresponds to a jump represented by the vertical black arrow. The clustered state (green) is now stable. We next ask what happens if locusts are now removed from the aggregate, which corresponds to a reduction in 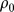 (moving to the left in Fig. 7). We answer this question numerically, by gradually subtracting mass from the population, allowing the system dynamics to evolve, and plotting the gregarious fraction as a function of mass. As the mass is slowly removed, the solution tracks leftwards along the green curve, indicating the persistence of the gregarious band. In fact, the band persists even partway into the regime where the HSS is linearly stable.

This dynamically observed hysteresis suggest that (for our model) a gregarious aggregation cannot be eliminated by reducing overall density to a low enough level where the HSS is linearly stable. This result has implications for locust control, as we discuss below.

## Discussion

In this paper, we derived, analyzed, and simulated a model for the movement, social interactions, and density-dependent interconversions of the solitarious and gregarious forms of phase polyphenic locusts. The model is based on experimental observations and measurements, parameter values inferred from preexisting work, and basic assumptions about individuals' rules of behavior. We included social exchanges via repulsive and/or attractive interactions for gregarious and solitarious individuals, and we accounted for phase change with density-dependent transitions, with crowding favoring solitarious-to-gregarious conversions. Our model was formulated in terms of continuum equations, allowing us to apply classical techniques such as linear stability analysis and bulk approximation. Since these methods were applied in two spatial dimensions, our results are relevant to insects aggregating in two dimensional structures such as hopper bands. We also provided example simulations in one spatial dimension as proof of principle, and as an indication of typical dynamics.

Our model explicitly takes into account intrinsic social interactions between individuals, in contrast to pre-existing models that focus on how insects respond to quality and spatial heterogeneity of nutrition or other environmental factors [8], [9], [24]. These approaches are complementary, showing that both intrinsic and extrinsic factors that affect local densities also affect the gregarization transition.

Many of our results are achieved via mathematical analysis. The power of mathematical analysis is that it creates an explicit connection between individual-level and group-level quantities, *e.g.*, via the inequality Eqn. (14). Once we identify the sensing range and interaction strength parameters in Eqn. (5) which govern individual locust attraction to and repulsion from others, we are able to calculate the critical density beyond which mass gregarization occurs.

Briefly, our results and predictions can be summarized as follows: (1) Locusts exist in a spatially uniform steady state distribution only up to a critical total population density. (2) Beyond this critical density, the uniform distribution can not be maintained, and massively dense gregarious clusters form. (3) Linear stability analysis allows us to understand how the critical density depends on dimensionless ratios of the biological parameters. This dependence is summarized in Fig. 2. Our analysis also yields the most unstable cluster spacing (from the wave number of the most unstable modes). (4) Numerical simulations illustrate the rapid transitions that take place once gregarization is initiated. Dense packs of gregarious locusts form and grow, and these move and sweep up solitarious locusts in their vicinity. (5) Via bulk approximation, we find estimates for the long-time mass fraction dynamics of solitarious and gregarious locusts. In the large population limit, the entire population will become gregarious. Bulk theory and simulations agree well, as shown in Fig. 6. (6) Our model displays population-level hysteresis, via which the critical density at which a gregarious aggregation forms from a dispersed population can be significantly higher than the density at which a gregarious aggregation would break up, as shown in Fig. 7.

Our results shed light on locust control strategies in two ways. First, given the mass gregarization that takes place past the point of linear instability, the density threshold for this instability is a crucial quantity. In accordance with the idea proposed in [42], our work identifies a threshold below which populations should be kept in order to avoid a gregarious outbreak (assuming biological parameters are known to a sufficiently accurate degree). Furthermore, we have shown how this population-level property depends on individual-level parameters, finding a nontrivial relationship. Second, the apparent population-level hysteresis shows that dispersing a gregarized band, perhaps by killing individuals with pesticides, is harder than preventing group formation in the first place in that band annihilation requires a significantly lower locust density. In short, hysteresis implies that prevention could be more easily achieved than control.

Like all models, ours has its limitations. We did not include features of the environment such as vegetation, shown to have important influence on local crowding and hence gregarization. Our simplifications lead to mathematical tractability, while limiting the direct biological relevance of the model at present. In the field, locusts encounter patchy vegetation and other environmental influences, and adding such factors to the model would make it more relevant to field experiments. Since we have not explicitly included resource gradients or other environmental cues, we do not here recapitulate the long-range motion of locust bands, but merely their formation and clustering. Including environmental factors constitutes an extension of the current framework. Similarly, simulations in two spatial dimensions are more challenging and remain open for future investigation.

Our work suggests several future biological experiments. First, as always, more accurate knowledge of model inputs would lead to better results. For our model, key inputs include the social interaction parameters, namely the length scales (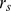, 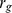, and 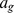) and interaction amplitudes (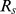, 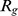, and 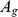) in Eqn. (5) that we inferred from careful experiments such as those in [24]. However, to our knowledge, most of these parameters have not been directly measured in experiments on individuals. Second, we encourage observations of macroscopic group properties that could be compared to outputs of our model. These outputs include densities and sizes of bands. Additional quantitative field measurements along the lines of [43] could help validate and refine our model. Finally, we can imagine experiments that would probe important aspects of the system dynamics (as opposed to physical properties of the bands themselves). Hopper bands are known to undergo complicated dynamics, including splitting and merging [3]. BBC video shows an example of such phenomena in *Locustana pardalina* bands [44]. More accurate data for the dynamics of wild groups, including times for group formation and distances between merging bands and tributaries, could be compared to clumping time and length scales identified by our model. We are especially curious about experiments in which the critical average density for population-level gregarization and clumping might be probed in a controlled lab experiment, perhaps by slowly adding solitarious individuals into a large arena. Experimental measurements like those we have mentioned here would also motivate future two-dimensional extensions of our model where the streaming dynamics of hopper bands, the effects of the environment, and other stimuli could be more fully explored.

## Supporting Information

Text S1 The Supporting Information (Text S1) provides technical details pertaining to parameter selection and estimation, the calculation of homogeneous steady states in our model, linear stability analysis, and the computational method used to carry out model simulations.
(PDF)
