# Supplementary material for: Locust Dynamics: Behavioral Phase Change and Swarming
Source: PLoS Comput Biol. 2012 Aug 16;8(8):e1002642. doi: 10.1371/journal.pcbi.1002642 (PMC3420939; doi:10.1371/journal.pcbi.1002642)
Supplement: Text S1 — The Supporting Information (Text S1) provides technical details pertaining to parameter selection and estimation, the calculation of homogeneous steady states in our model, linear stability analysis, and the computational method used to carry out model simulations. (PDF) [file pcbi.1002642.s001.pdf]

Supporting Information

Chad M. Topaz[1,*], Maria R. D'Orsogna[2], Leah Edelstein-Keshet[3] Andrew J. Bernoff[4]
1 Department of Mathematics, Statistics, and Computer Science, Macalester College, Saint Paul, Minnesota, United States of America
2 Department of Mathematics, California State University at Northridge, Los Angeles, California, United States of America
3 Department of Mathematics, University of British Columbia, Vancouver, British Columbia, Canada
4 Department of Mathematics, Harvey Mudd College, Claremont, California, United States of America
∗ **E-mail: ctopaz@macalester.edu**

Model equations

For convenience, we reintroduce our model equations. Consider a two-dimensional domain Ω with spatial coordinate $\mathbf{x} = (x, y)$. Define $\rho(\mathbf{x}, t) = s(\mathbf{x}, t) + g(\mathbf{x}, t)$ as the locust population density field, with $s(\mathbf{x}, t)$ and $g(\mathbf{x}, t)$ the solitary and gregarious components, respectively. The locust populations move with velocities $\mathbf{v}_{s,g}(\mathbf{x}, t)$ and obey the equations

$$\dot{s} + \nabla \cdot (\mathbf{v}_s s) = -f_2(\rho)s + f_1(\rho)g, \quad \mathbf{v}_s = -\nabla(Q_s * \rho), \tag{1a}$$

$$\dot{g} + \nabla \cdot (\mathbf{v}_g g) = f_2(\rho)s - f_1(\rho)g, \quad \mathbf{v}_g = -\nabla(Q_g * \rho), \tag{1b}$$

These equations generalize the classic swarming model

$$\rho_t + \nabla \cdot (\rho \mathbf{v}) = 0, \quad \mathbf{v} = -\int_\Omega \nabla Q(\mathbf{x} - \mathbf{x}')\rho(\mathbf{x}', t)d\mathbf{x}', \tag{2}$$

which describes a single population density field advected by a velocity field arising from social interactions. Eq. (2) has been studied extensively in one and two spatial dimensions for various social interaction functions represented by Q, whose negative gradient is the effective social force [1–4]. Depending on Q, solutions include steady swarms, spreading populations, and contracting groups (*i.e.*, blow-up) [2, 5, 6].

In our two-phase model Eqs. (1), the velocities are

$$\mathbf{v}_{s,g}(\mathbf{x}, t) = -\nabla Q_{s,g} * \rho \equiv -\int_\Omega \nabla Q_{s,g}(\mathbf{x} - \mathbf{x}')\rho(\mathbf{x}', t)\, d\mathbf{x}', \tag{3}$$

and the social interaction potentials $Q_{s,g}$ are

$$Q_s(\mathbf{x} - \mathbf{x}') = R_s \mathrm{e}^{-|\mathbf{x}-\mathbf{x}'|/r_s}, \quad Q_g(\mathbf{x} - \mathbf{x}') = R_g \mathrm{e}^{-|\mathbf{x}-\mathbf{x}'|/r_g} - A_g \mathrm{e}^{-|\mathbf{x}-\mathbf{x}'|/a_g}. \tag{4}$$

Here, R_s, R_g, A_g are interaction magnitudes and r_s, r_g and a_g are interaction length scales. We require $R_g a_g - A_g r_g > 0$ and $A_g a_g^2 - R_g r_g^2 > 0$ so that Q_g includes short range repulsion and long range attraction, as in [5–7], as this is the clumping regime, appropriate to capture the tendency of gregarious locusts to aggregate. We model the density-dependent rates of interconversion of the solitary and gregarious forms as

$$f_1(\rho) = \frac{\delta_1}{1 + (\rho/k_1)^2}, \quad f_2(\rho) = \frac{\delta_2\,(\rho/k_2)^2}{1 + (\rho/k_2)^2}. \tag{5}$$

The parameters $\delta_{1,2}$ are maximal rates and $k_{1,2}$ are characteristic locust densities at which the transitions occur at half of their maximal values. To the best of our knowledge, our work is the first to consider locust phase changes via continuum modeling of locust density [1–4].

Parameter selection and estimation

As discussed in the main text, for our numerical results, we use two different sets of phase change parameters. For both sets, we use the same social interactions parameters, and we now describe our choices for these.

To estimate R_s, R_g, and A_g, we use explicit velocity computations. The speed of a locust when it is alone varies between 72 - 216 m/hr, while the speed of a locust in a group varies in a tighter range of 144 - 216 m/hr [8]. To make a rough estimate of R_s, we imagine a hypothetical semi-infinite density field $\rho(x,y) = \rho_{group}\mathrm{H}(x)$ where $\mathrm{H}(x)$ is the Heaviside function and, as mentioned in the main text, $\rho_{group} = 65\ locusts/m^2$ is the approximate critical density of a gregarious group [9]. A solitary locust placed at the swarm's edge (at the origin) should move to the left with maximal velocity $v_s^{\max} = -216\ m/hr$. From Eqn. (3),

$$v_s(0,0) = \{-\nabla Q_s * \rho_{group}\mathrm{H}(x)\}\,\big|_{(0,0)} = v_s^{\max}, \tag{6}$$

which we solve to find $R_s = 11.87\ m^3/(hr \cdot locust)$. Similarly, a gregarious locust at the origin should move to the right with maximal velocity $v_g^{\max} = 216\ m/hr$, so

$$v_g(0,0) = \{-\nabla Q_g * \rho_{group}\mathrm{H}(x)\}\,\big|_{(0,0)} = v_g^{\max}. \tag{7}$$

A gregarious locust placed to the left of the swarm at a distance equal to the attraction length scale $a_g = 0.14\ m$ should also move to the right, but with a slower velocity which we take to be the minimal velocity in a crowd, $v_g^{\min} = 144\ m/hr$. Thus

$$v_g(-0.14,0) = \{-\nabla Q_g * \rho_{group}\mathrm{H}(x)\}\,\big|_{(-0.14,0)} = v_g^{\min}. \tag{8}$$

These two conditions determine $R_g = 5.13\ m^3/(hr \cdot locust)$ and $A_g = 13.33\ m^3/(hr \cdot locust)$ In the main text, we present numerical simulations of Eqs. (1) in one spatial dimension. For these simulations, we take $\delta_{1,2}$, r_s, r_g, and a_g as above, since these parameters do not depend on spatial dimension. For the remaining parameters, we follow a process similar to that described above, and choose $k_{1,2} = k = 8\ locusts/m$, $R_s = 6.83\ m^2/(hr \cdot locust)$, $R_g = 6.04\ m^2/(hr \cdot locust)$, and $A_g = 12.9\ m^2/(hr \cdot locust)$.

Homogeneous steady states

For any set of initial conditions, the mean locust density ρ_0 is known, and corresponds to the total density at the homogeneous steady state (HSS). Accordingly, there is a family of homogeneous steady states parameterized by ρ_0. The corresponding solitary and gregarious HSS components, obtained by setting time and space derivatives to zero in Eqs. (1) are

$$s_0 = \frac{\rho_0\delta_1 k_1^2(k_2^2+\rho_0^2)}{\delta_1 k_1^2 k_2^2 + \delta_1 k_1^2\rho_0^2 + \delta_2 k_1^2\rho_0^2 + \delta_2\rho_0^4}, \tag{9a}$$

$$g_0 = \frac{\delta_2\rho_0^3(k_1^2+\rho_0^2)}{\delta_1 k_1^2 k_2^2 + \delta_1 k_1^2\rho_0^2 + \delta_2 k_1^2\rho_0^2 + \delta_2\rho_0^4}. \tag{9b}$$

When we later consider stability of homogeneous steady states, it will be convenient to discuss the fractions $\phi_{s,g}$ of solitarious and gregarious locusts, where $\phi_s + \phi_g = 1$. Using Eqn. (9), we know that for homogeneous steady states,

$$\phi_g = \frac{g_0}{s_0+g_0}, \tag{10a}$$

$$= \frac{1}{s_0/g_0+1}, \tag{10b}$$

$$= \left\{1 + \gamma K^2\frac{1+\psi^2}{\psi^2(\psi^2+K^2)}\right\}^{-1}. \tag{10c}$$

Here, $\gamma = \delta_1/\delta_2$ is the ratio of maximal solitarization rate to maximal gregarization rate, $K = k_1/k_2$ is the ratio of the characteristic solitarization and gregarization densities for individuals, and $\psi = \rho_0/k_2$ is a rescaled density. Note that ϕ_g is monotonically increasing in ψ, and hence in ρ_0; that is to say, as total density increases, the gregarious fraction increases.

Linear stability analysis

To study the stability of the HSS in Eqs. (9), we consider small perturbations s_1, g_1 about s_0, g_0

$$s(\mathbf{x}, t) = s_0 + s_1(\mathbf{x}, t), \quad g(\mathbf{x}, t) = g_0 + g_1(\mathbf{x}, t), \tag{11}$$

so that $\rho(\mathbf{x}, t) = s_0 + g_0 + s_1(\mathbf{x}, t) + g_1(\mathbf{x}, t)$. Substituting Eqn. (11) into Eqn. (1) and expanding to first order in the perturbations, we find the linearized equations

$$\dot{s}_1 = s_0 Q_s * \nabla^2(s_1 + g_1) - A s_1 + B g_1, \tag{12a}$$
$$\dot{g}_1 = g_0 Q_g * \nabla^2(s_1 + g_1) + A s_1 - B g_1, \tag{12b}$$

where

$$A = f_2(\rho_0) + f_2'(\rho_0) s_0 - f_1'(\rho_0) g_0, \tag{13a}$$
$$B = f_1(\rho_0) + f_1'(\rho_0) g_0 - f_2'(\rho_0) s_0. \tag{13b}$$

Here, $A, B > 0$ for all $\rho_0 > 0$ since f_1 is a monotonically increasing function of ρ_0 and f_2 is a monotonically decreasing one. To further analyze the linearized equations, we Fourier expand the perturbations as

$$s_1(\mathbf{x}, t) = \sum_{\mathbf{q}} \mathcal{S}_{\mathbf{q}}(t) \mathrm{e}^{i\mathbf{q}\cdot\mathbf{x}}, \quad s_2(\mathbf{x}, t) = \sum_{\mathbf{q}} \mathcal{G}_{\mathbf{q}}(t) \mathrm{e}^{i\mathbf{q}\cdot\mathbf{x}}. \tag{14}$$

We allow for an infinitely large domain so that there are no restrictions on $\mathbf{q}$; in other situations, $\mathbf{q}$ must be suitably restricted in order to satisfy boundary conditions. Substituting Eqn. (14) into Eqn. (12) yields ordinary differential equations for each Fourier mode amplitude. We write these in matrix form,

$$\frac{d}{dt}\begin{pmatrix} \mathcal{S}_q \\ \mathcal{G}_q \end{pmatrix} = \mathbf{L}(q) \begin{pmatrix} \mathcal{S}_q \\ \mathcal{G}_q \end{pmatrix}, \tag{15a}$$

$$\mathbf{L}(q) \equiv \begin{pmatrix} -s_0 q^2 \widehat{Q}_s(q) - A & -s_0 q^2 \widehat{Q}_s(q) + B \\ -g_0 q^2 \widehat{Q}_g(q) + A & -g_0 q^2 \widehat{Q}_g(q) - B \end{pmatrix}. \tag{15b}$$

Here, $q = |\mathbf{q}|$ is the perturbation wavenumber, and $\widehat{Q}_{s,g}(q)$ are the Fourier transforms of the two dimensional social interaction potentials,

$$\widehat{Q}_s(q) = \frac{2\pi R_s r_s^2}{(1 + r_s^2 q^2)^{3/2}}, \tag{16}$$

$$\widehat{Q}_g(q) = \frac{2\pi R_g r_g^2}{(1 + r_g^2 q^2)^{3/2}} - \frac{2\pi A_g a_g^2}{(1 + a_g^2 q^2)^{3/2}}. \tag{17}$$

The eigenvalues $\lambda_{1,2}(q)$ of $\mathbf{L}(q)$ are

$$\lambda_1(q) = -q^2 \left[s_0 \widehat{Q}_s(q) + g_0 \widehat{Q}_g(q) \right], \quad \lambda_2 = -(A + B). \tag{18}$$

Since $\lambda_2 < 0$, instability occurs only when $\lambda_1 > 0$. For convenience, we rewrite λ_1 in terms of the gregarious mass fraction ϕ_g,

$$\lambda_1(q) = -\rho_0 q^2 \left[(1-\phi_g)\widehat{Q}_s(q) + \phi_g \widehat{Q}_g(q)\right]. \tag{19}$$

Now we factor out the attractive part of the gregarious term, namely

$$\phi_g \frac{2\pi A_g a_g^2}{(1+a_g^2 q^2)^{3/2}}. \tag{20}$$

This yields

$$\lambda_1(q) = -\rho_0 q^2 \phi_g \frac{2\pi A_g a_g^2}{(1+a_g^2 q^2)^{3/2}} \left[\frac{1-\phi_g}{\phi_g}\frac{R_s r_s^2}{A_g a_g^2}\frac{(1+a_g^2 q^2)^{3/2}}{(1+r_s^2 q^2)^{3/2}} + \frac{R_g r_g^2}{A_g a_g^2}\frac{(1+a_g^2 q^2)^{3/2}}{(1+r_g^2 q^2)^{3/2}} - 1\right]. \tag{21}$$

Since the prefactor is negative, and we seek conditions for a positive eigenvalue (signifying growth of perturbations, and hence instability), we focus on when the term in square brackets becomes negative. The dependence on ϕ_g occurs via the prefactor $(1-\phi_g)/\phi_g$ in front of a positive term. For possible instability, this term should be small, meaning that ϕ_g should be sufficiently large (since this prefactor is monotonically decreasing with ϕ_g). Since ϕ_g increases monotonically with ρ_0 (as discussed above), instability may occur as ρ_0 is increased.

We now show that instability first occurs at the wavenumber $q = 0$ (meaning that perturbations that first lead to instability are long wavelength). We again focus on the bracketed quantity in Eq. (21). If this term becomes negative, it must do so for the value of q at which the first two terms are (together) minimized, since these are positive terms and the negative term, -1, is a constant. It is biologically reasonable to assume that $a_g \geq r_s$ (with equality achieved for our chosen social interaction parameters). Therefore, the first term is either constant or monotonically increasing in q. It is also biologically reasonable to assume that $a_g > r_g$, in which case the second term is monotonically increasing in q. Thus, the first two terms together are monotonically increasing in q, so their minimum occurs at $q = 0$, and this will be the first wavenumber to trigger instability. Thus, if we are looking for the instability that occurs as ϕ_g increases, it is sufficient to consider what happens at $q = 0$.

We substitute $q = 0$ into the bracketed term in Eqn. (21) and ask for what value of ϕ_g the resultant expression changes sign (to find the threshold level of gregarious locust fraction needed for instability). Setting that bracketed term to zero we obtain

$$\phi_g^* = \frac{R_s r_s^2}{R_s r_s^2 - R_g r_g^2 + A_g a_g^2}. \tag{22}$$

Instability is achieved for values of ϕ_g greater than this threshold value.

To obtain a more explicit condition for instability in terms of the density ρ_0, we substitute ϕ_g^* into Eq. (10), which relates gregarious fraction to total (scaled) density. Rearranging, we obtain the biquadratic equation

$$A\psi^4 + B\psi^2 + C = 0, \tag{23}$$

where

$$A = \frac{1}{\phi_g^*} - 1, \tag{24a}$$

$$B = K^2\left(\frac{1}{\phi_g^*} - 1 - \gamma\right), \tag{24b}$$

$$C = -\gamma K^2. \tag{24c}$$

For any biologically meaningful solutions, the solution for ψ^2 must be positive. From the quadratic formula, we have

$$\psi^2 = \frac{-B \pm \sqrt{B^2 - 4AC}}{2A}. \tag{25}$$

Since $A > 0$ and $C < 0$, the discriminant is positive. Hence, for the plus sign choice, $\psi^2 > 0$. For the minus sign choice, $\psi^2 < 0$ and hence we eliminate this possibility. The final result for the critical scaled density is

$$\psi^* = \sqrt{\frac{-B + \sqrt{B^2 - 4AC}}{2A}}. \tag{26}$$

This is the result that we use to produce instability contours in the K-γ plane (Fig. 2 in the main paper).

Numerical simulation method

We simulate Eqs. (1)-(5) in one spatial dimension. We use periodic boundary conditions on a domain of length L with a fine grid consisting of $N = 1024$ points (necessary to resolve the steep edges of clusters that form). To approximate an unbounded domain, one may take the limit of large L. The social interactions $Q_{s,g}$ in (4) must be adapted to be commensurate with a periodic domain. We begin with the function $Q(x) = \mathrm{e}^{-|x|/r}$, which is the building block of $Q_{s,g}$. We calculate the discrete Fourier transform $\mathcal{F}$ of $-\partial_x Q$ on our domain as

$$\mathcal{F}\{-\partial_x Q(x)\} = -\frac{i}{r}\frac{\Delta \sin(\Delta q)}{\cosh(\Delta/r) - \cos(\Delta q)}, \tag{27}$$

where r is the decay length scale in Q and $\Delta = L/N$ is the grid spacing. From Eqn. (27) it is straightforward to compute the Fourier transforms of $Q_{s,g}$. Convolutions are equivalent to products in Fourier space, providing excellent computational savings (and thus justifying the choice of a periodic domain). We compute velocities by convoluting the density with $-\partial_x Q_{s,g}$ pseudospectrally. The flux term in Eqs. (1) is instead evaluated via a fourth-order accurate central finite difference.

The emergence of discontinuities in s and g causes ringing in the pseudospectral evaluation of the velocity term. In order to smooth this effect, we incorporate small amounts of numerical diffusion. Another standard approach would be to incorporate high wave number filtering in the simulation. We choose numerical diffusion because it also serves as the macroscopic description of random motion, which locusts certainly display. We implement diffusion in a split-step manner, alternating with the dynamics of Eqs. (1)-(5). Time-stepping is performed with the fourth-order Runge-Kutta method. We also threshold our velocity field at every time step so that it does not exceed $v_g^{\max}$. Without this thresholding, individual locusts achieve velocities of up to approximately 1.5 times $v_g^{\max}$ at an intermediate stage of our simulation. It is crucial to point out that this thresholding only affects the speed of the transient clumps; it does not affect the initial instability (which is small amplitude, and thus has a small velocity) and similarly, it does not affect the late-stage bulk dynamics (which are nearly spatially stationary).

References

1. Topaz CM, Bertozzi AL (2004) Swarming patterns in a two-dimensional kinematic model for biological groups. SIAM J Appl Math 65: 152-174.

2. Bertozzi AL, Laurent T (2007) Finite-time blow-up of solutions of an aggregation equation in $\mathbb{R}^n$. Comm Math Phys 274: 717-735.

3. Bodnar M, Velasquez JJ (2005) Derivation of macroscopic equations for individual cell-based models: A formal approach. Math Meth Appl Sci 28: 1757-1779.

4. Bodnar M, Velasquez JJ (2006) An integro-differential equation arising as a limit of individual cell-based models. J Diff Eq 222: 341-380.

5. Leverentz AJ, Topaz CM, Bernoff AJ (2009) Asymptotic dynamics of attractive-repulsive swarms. SIAM J Appl Dyn Sys 8: 880-908.

6. Bernoff AJ, Topaz CM (2011) A primer of swarm equilibria. SIAM J Appl Dyn Sys 10: 212-250.

7. Chuang YL, D'Orsogna MR, Marthaler D, Bertozzi AL, Chayes LS (2007) State transitions and the continuum limit for a 2D interacting, self-propelled particle system. Physica D 232: 33-47.

8. Bazazi S, Romanczuk P, Thomas S, Schimansky-Geier L, Hale JJ, et al. (2011) Nutritional state and collective motion: From individuals to mass migration. Proc Roy Soc B 278: 356-363.

9. Simpson SJ, Despland E, Hägele BF, Dodgson T (2001) Gregarious behavior in desert locusts is evoked by touching their back legs. Proc Natl Acad Sci 98: 3895-3897.
